# Management of Early Post-Transplant Hyperglycemia by Dedicated Endocrine Care Improves Glycemic Outcomes

**DOI:** 10.3390/clinpract14050156

**Published:** 2024-09-25

**Authors:** Alon Kaplan, Tslil Manela, Tammy Hod, Ronen Ghinea, Eytan Mor, Amit Tirosh, Amir Tirosh, Gadi Shlomai

**Affiliations:** 1 Internal Medicine D and the Hypertension Unit, Sheba Medical Center, Tel-Hashomer 52621, Israel; alonkaplan47@gmail.com (A.K.); tslilmanela@gmail.com (T.M.); 2 Tel Aviv Faculty of Medicine, Tel-Aviv University, Tel-Aviv 52621, Israel; amit.tirosh@sheba.health.gov.il (A.T.); amir.tirosh@sheba.health.gov.il (A.T.); 3 Renal Transplant Center, Sheba Medical Center, Tel-Hashomer 52621, Israel; tamar.hod@sheba.health.gov.il (T.H.); ronen.ghinea@sheba.health.gov.il (R.G.); eytan.mor@sheba.health.gov.il (E.M.); 4 Nephrology Department, Sheba Medical Center, Tel-Hashomer 52621, Israel; 5 Department of Surgery B, Sheba Medical Center, Tel-Hashomer 52621, Israel; 6 The Institute of Endocrinology, Diabetes and Metabolism, Sheba Medical Center, Tel-Hashomer 52621, Israel; 7 ENTIRE—Endocrine Neoplasia Translational Research Center, Research Center for Endocrinology, Diabetes and Metabolism, Tel-Hashomer 52621, Israel

**Keywords:** post-transplant hyperglycemia, dedicated endocrine care, glycemic control

## Abstract

Introduction: Early post-transplant hyperglycemia (EPTH) is an independent risk factor for hospital readmissions, acute rejection, infections and developing post-transplant diabetes mellitus (PTDM). Close glycemic control is prudent in the early post-transplant period. The management of EPTH was evaluated among a cohort of kidney transplant recipients, who either received routine care (RC) or dedicated endocrine care (DEC). Methods: A retrospective analysis was conducted on kidney transplant recipients from 2019 to 2023. The impact of DEC on post-transplant glycemic control was investigated. Hospitalized patients receiving post-transplant insulin therapy were included. DEC involved at least twice-daily blood glucose (BG) assessment by an endocrinologist, while the RC received usual care. A mixed-model analysis was employed to assess differences in BG trajectories between DEC and RC over an eight-day period. Additionally, various glycemic control metrics were compared, including glucose variability, time-in-range for target BG, rates of hypoglycemia and response to hyperglycemia. Results: The cohort comprised 113 patients. In the DEC group, 91% had pre-transplant DM compared to 15% in the RC group (*p* < 0.001). Patients under DEC had higher baseline BG and glycated hemoglobin compared to those under RC (*p* < 0.001, for both). The DEC group displayed a lower trajectory of BG over time compared to the RC group (*p* = 0.002). Patients under DEC were more likely to receive insulin if BG measured above 200 mg/dL (66% vs. 46%) and displayed less below-range BG (<110 mg/dL) compared to those under RC (12.9% vs. 23.6%, *p* < 0.001). Conclusions: Management of EPTH by DEC improves glycemic outcomes in renal transplant recipients.

## 1. Introduction 

Diabetes mellitus (DM) is an overwhelming worldwide public health issue [1]. Approximately 40% of patients with DM will experience renal involvement, and it is the leading cause of end-stage renal disease [1], constituting 30% of renal transplant candidates [2]. 

Hyperglycemia is very common during hospitalization in the early post-transplant setting [3]. Immediately post-transplant, random blood glucose (BG) measurements above 200 mg/dL or insulin requirement are defined as early in-hospital post-transplant hyperglycemia (EPTH), and this requires insulin treatment and close monitoring [4]. All patients with pre-existing DM and 87% of those without prior DM show evidence of in-hospital hyperglycemia during the immediate post-transplant period, and 66% of those without pre-existing DM have been shown to require insulin upon discharge [3]. Risk factors for post-transplant hyperglycemia include pre-transplant glucose intolerance and frank DM [5,6], use of immunosuppressant drugs, particularly high-dose IV steroids [7,8,9,10], surgical stress, postoperative pain and infections. Due to the high frequency and potential reversibility of immediate post-transplant hyperglycemia, the 2013 Vienna Consensus Meeting concluded that a diagnosis of post-transplant DM (PTDM) can only be made 45 days post-transplant [11], and hyperglycemia during the initial 45 days is thereby termed EPTH [4]. While EPTH has been less studied than PTDM, it is an independent risk factor for hospital readmissions [12], worse renal graft function and acute rejection [13,14,15,16,17,18], higher proteinuria and chronic nephropathy [14], a higher risk for infections [13,14] and future PTDM development [5,14,18,19]. 

Basal–bolus insulin treatment remains the standard-of-care for managing in-hospital EPTH. The American Diabetes Association (ADA) recommends a target BG of 140–180 mg/dL for most ill and non-critically ill patients [20] and a more stringent goal of 110–140 mg/dL in selected patients, if hypoglycemia can be avoided [20]. Thus, closely monitoring the development of in-hospital hyperglycemia and hypoglycemia is prudent in the immediate post-transplant period. 

Therefore, the management of EPTH and glycemic outcomes among a cohort of renal transplant recipients, who either received daily routine care (RC) by the transplant team staff or dedicated endocrine care (DEC) by an endocrinologist, was evaluated. 

## 2. Methods

### 2.1. Study Design

This retrospective study included kidney transplant recipients admitted to the Sheba Medical Center, Israel, between 2019 and 2023. Following the definitions of EPTH, which were recently described [4], all patients who were administered insulin at least once during their post-transplant hospitalization and irrespective of their pre-transplant glycemic status, were defined as EPTH and included in the analysis. Patients were categorized into two groups: those receiving routine care (RC) and those receiving dedicated endocrine care (DEC). DEC included in-hospital diabetes education by a physician and nurse practitioner and BG evaluation at least twice daily and insulin dose titration accordingly. Routine care (RC) included daily visits by the transplant team and insulin treatment and titration at their discretion. DEC was initiated in January 2020, while RC was implemented in early 2019. No additional exclusion criteria were applied. 

### 2.2. Immunosuppressive Protocol

The immunosuppressive protocol was based on induction therapy using thymoglobulin (Genzyme, Boston, MA, USA) 1.5 mg/kg in three divided doses for sensitized patients with preformed anti-HLA Ab’s and for re-transplantation, whereas Simulect (Novartis Pharma, Basel, Switzerland), 20 mg per dose at days 1 and 4 post-transplant, was administrated for first transplant non-sensitized patients. Maintenance immunosuppression was based on a combination of tacrolimus and myfortic (mycophenolic acid, Novartis, Switzerland) with a tapered glucocorticoid dose. For low immunological risk transplant patients, glucocorticoid was discontinued at days 6–8 post-transplant [21,22], whereas for all other patients, steroid therapy was maintained [23]. 

The Sheba Medical Center Helsinki Committee (approval number 0504-23) approved this study. Data were recorded anonymously. No individual consent was obtained.

### 2.3. Statistical Analysis

Baseline characteristics were expressed as means (standard deviations [SDs]), medians (interquartile ranges [IQRs]), or numbers (percentages). Normality was assessed using the Shapiro–Wilk test and visual histograms. Student’s *t*-tests and Mann–Whitney U tests were employed for comparisons, depending on the normal distribution of continuous variables. Categorical variables were compared using the Pearson chi-square test or Fisher’s exact test.

The primary endpoint was BG trajectory post-transplant, with secondary endpoints encompassing rates of hypoglycemic events, glucose levels within the accepted range, hyperglycemic insulin response, daily insulin, glucose coefficient of variation (CV) and short-term complications such as 30-day readmission, infections, graft loss and acute rejections. 

The differences in BG between participants who received DEC and those who received RC were investigated. A mixed model analysis was employed. This method was selected due to the substantial between-group baseline differences in glycemic indices, such as fasting blood glucose, hemoglobin A1c and diabetes complications. This statistical approach analyzes repeated BG measurements (five per day over eight days) and accounts for individual variations in BG patterns (random effect). The analysis allows for isolation of the specific effect of DEC on the BG trajectory. Due to the limited sample size, further confounder adjustments were not feasible. A scatter plot was produced with the serum glucose consecutive measurement number and test values in axes x and y, respectively. Plots were produced using ggplot2 [24] in R statistical software version 4.4.0 (R Core Team (2021)). The loess method was used for a smooth regression line with a 95% confidence interval. Secondary endpoints were analyzed using appropriate statistical tests, as mentioned above. 

Given the exploratory nature of this study, a formal power calculation was not conducted prior to data collection. However, to provide a reference point, we estimated the required sample size based on a previous study [6]. This study assessed the difference in early posttransplant hyperglycemia between patients with diabetes mellitus and those who developed posttransplant diabetes mellitus within the first day after transplantation. Using the sample size of our study, a significance level of 5% and a desired effect size of 68.4 mg/dL (3.8 nnmol/L) in blood glucose level, a power calculation revealed 100% power to detect such a difference.

All P-values were two-sided, and significance was set at <0.05. IBM SPSS Statistics version 29 and GraphPad Prism 9 were used for data analysis and graph creation, respectively.

## 3. Results

206 patients underwent renal transplant in our institution during the study period. Of them, 113 received insulin at least once during post-transplant hospitalization and, therefore, were included in this study. The mean age was 58.2 ± 11.8 years, and 71.7% were men. The mean fasting BG level was 141.4 ± 64.1 mg/dL, mean HbA1c was 6.1 ± 1.3% and half of the participants had pre-transplant diabetes (Table 1). 

Compared to the RC group, patients in the DEC group were older (*p* = 0.009), were more likely to have pre-transplant diabetes (*p* < 0.01) and diabetes-related microvascular complications (*p* < 0.01), as well as higher rates of ischemic heart disease (*p* = 0.02). They also had higher baseline BG levels (*p* < 0.01) and HbA1c levels (*p* < 0.01) (Table 1).

**Figure 1:**
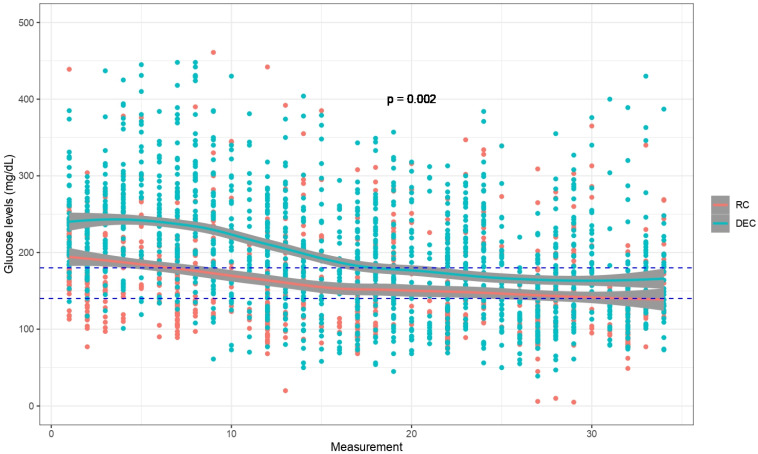
Blood glucose trajectory during post-transplant hospitalization: glucose levels were measured five times a day. Scatter plot of the serum glucose levels by consecutive measurement, compared between RC and DEC. Dashed lines denote the target serum glucose levels (140–180 mg/dL), and the continuous lines indicate regression lines, with gray shadowing denoting a 95% confidence interval.

A lower trajectory of BG over time was observed in the DEC group compared to the RC group (*p* = 0.002) (Figure 1). The DEC group also had lower rates of below-range BG values, defined as BG less than 110 mg/dL (12.9% vs. 23.6%, *p* = 0.02), while rates of BG less than 70 mg/dL were rare overall and comparable between groups (1.3%, *p* = 0.6, Table 2). In addition, short-term transplant-related complication rates, such as 30-day readmission (20.8% vs. 23.3%, *p* = 0.74, for DEC and RC, respectively), infections (26.4% vs. 28.3%, *p* = 0.82, for DEC and RC, respectively), rejection (11.3% vs. 16.7%, *p* = 0.42, for DEC and RC, respectively) and graft loss (3.8% vs. 8.3%, *p* = 0.44, for DEC and RC, respectively) were statistically similar between groups (Table 3). 

**Figure 2:**
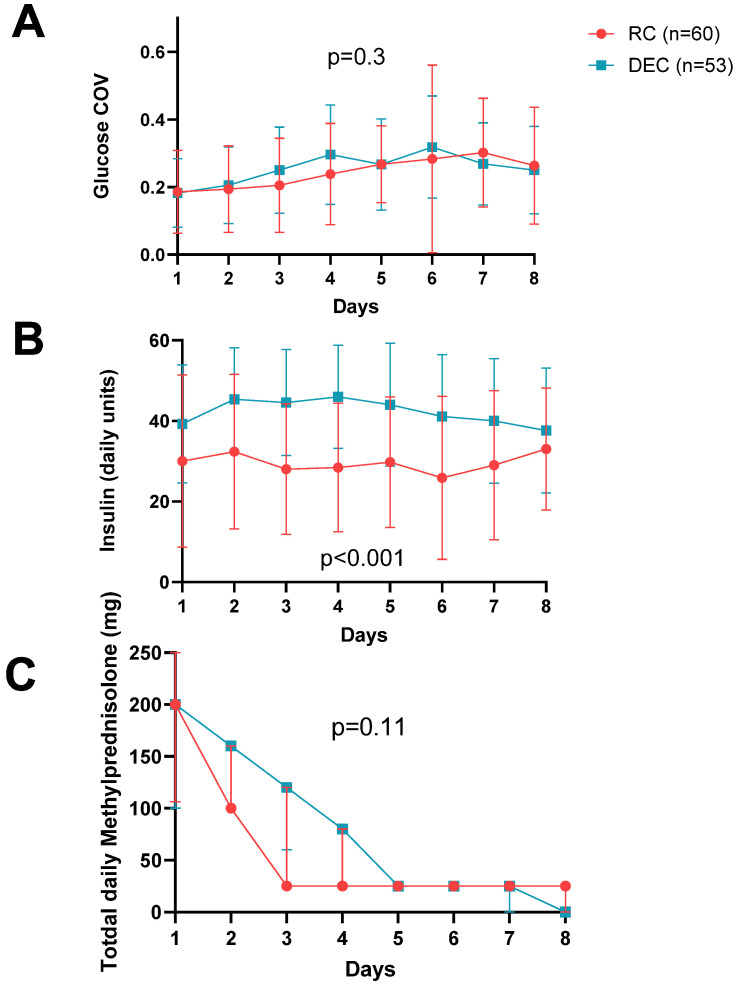
Insulin administration, steroid dose, and glycemic control during post-transplant hospitalization: (**A**) the mean coefficient of variation in glucose levels (COV), calculated as the ratio of the standard deviation to the mean of daily glucose levels. (**B**) The mean daily insulin unit dosage. (**C**) The median daily steroid dosage in mg of methylprednisolone was overall similar but significantly higher in the DEC group on days 2–4 compared to the RC group (*p* < 0.05), presented as medians and IQR.

Although the DEC group received higher doses of glucocorticoids on three out of eight days (Table 4, *p* < 0.05) and a higher mean total daily insulin dose (42.8 ± 9 units vs. 27.6 ± 14.9 units, *p* < 0.001, Figure 2), the glucose coefficient of variation (COV) and BG time in range (TIR) were similar between groups, (*p* > 0.05; Figure 2 and Table 2 and Table 5). Additionally, the DEC group also had significantly higher rates of insulin response to hyperglycemia, i.e., administration of short-acting insulin when BG ≥ 200 mg/dL (66.4% vs. 46.3%, *p* < 0.001) (Table 2) with a comparable insulin sliding scale between groups. 

## 4. Discussion

In this study, we demonstrate that in kidney recipients, dedicated endocrine care (DEC) is associated with improved glycemic outcomes compared to routine care (RC). The data suggest that even though patients under DEC were significantly older, had higher rates of pre-transplant diabetes, elevated fasting glucose and glycated hemoglobin, as well as receiving higher doses of intravenous glucocorticoids, they manifested a significantly lower BG trajectory over time, which suggests superior inpatient diabetes care. 

EPTH is a very common complication within the first week following renal transplant [3,4,14], occurring in the vast majority of transplant patients with or without a history of pre-transplant diabetes [3,5,25]. It is variably defined as random BG above 200 mg/dL in the inpatient setting or insulin requirements any time after surgery [4]. Accordingly, for this study, EPTH was defined as any insulin requirement during the first eight days, post-transplant. 

EPTH and post-transplant glucose control are important risk factors for hospital readmissions, infections and acute rejection [12,13,14,16,17,18,19]. Furthermore, EPTH during the first week post-surgery has been shown to be the strongest predictor of future PTDM at one year [5,14,18,19], highlighting the importance of early insulin initiation. In this cohort, pre-transplant DM was noted for almost 91% of patients in the DEC group compared to only 15% of the RC population. Therefore, a comparison of PTDM rates six months post-transplant was not performed. 

Interestingly, rapid corticosteroid withdraw protocols are implemented widely for patients with low immunological profiles. However, data regarding glycemic outcomes are variable. In one study, rapid withdrawal did not influence insulin sensitivity significantly in a study utilizing hyperinsulinemic euglycemic glucose clamps [26]. However, a systematic review and meta-analysis of RCTs found that rapid steroid withdrawal was associated with less frequent PTDM, and in an open-label, multi-centric RCT, rapid steroid withdrawal, in patients with low immunological risk profiles, significantly reduced rates of PTDM [21,22]. Some of the patients in the current cohort underwent rapid corticosteroid withdrawal; however, the possible benefit would likely be reflected later in the post hospitalization phase, and, therefore, a subgroup analysis was irrelevant for the aims of this study and, thus, was not conducted. 

During the early post-transplant phase, early initiation of insulin is prudent, as evident by a randomized proof-of-concept clinical trial, which showed that early initiation of subcutaneous insulin during the immediate postoperative period significantly reduced the odds for future PTDM [25]. Indeed, the data show that, compared to those under RC, patients under DEC received significantly more rapid acting insulin in response to hyperglycemia, specifically in cases when BG measured over 200 mg/dL. One possible explanation for these findings is that there could be differences in the insulin sliding scales that raises the question of treatment bias. However, the insulin sliding scales were identical between groups; thus, this reduces the possible contribution of treatment bias and highlights the advantage of close and stringent monitoring by DEC. 

Renal transplant patients are sensitive to glycemic variability (i.e., hyper- and hypoglycemia). Studies have shown that among renal transplant recipients, glycemic variability and, specifically, hypoglycemia are well-established risk factors for in-hospital complications, such as falls [27] and renal failure [28]. In this study, while patients under DEC had significantly higher baseline BG and received higher IV steroid doses and higher total daily insulin doses compared to patients under RC, glucose variability portrayed by the CV value was comparable, and TIR (i.e., 140–180 mg/dL) was also similar between groups. In addition, patients under DEC had significantly lower rates of BG less than 110 mg/dL, while the frequency rates for BG less than 70 mg/dL were comparable and low in both groups. The ADA recommends a BG target of 140–180 mg/dL in non-critically ill patients, with a lower range in selected patients [20]. A randomized controlled trial assessing a tighter glycemic range of 70–110 mg/dL among renal transplant patients showed that intensive control was associated with significantly higher rates of in-hospital hypoglycemia and rejection [29]. Notably, while patients under DEC were older, had higher rates of pre-transplant diabetes and higher baseline BG, as well as more diabetes-related microvascular complications and ischemic heart disease, no significant difference in short-term transplant-related complications such as 30-day readmission rates, infections, rejections and graft loss were observed. These findings further strengthen the advantage of DEC in post-transplant care.

The study has several limitations, including its retrospective nature and reflecting the experience of a single center. The main limitation of this study is the heterogeneity of the cohort and, specifically, the significant difference in baseline characteristics between the two groups, as expected, which raises concerns for selection bias. However, patients’ allocation to each treatment group was arbitrary; DEC was available and implemented since January 2020, once the endocrine service could provide a dedicated endocrinologist to follow up with hospitalized kidney recipients. Therefore, all patients who were admitted prior to January 2020, i.e., since initiating our kidney transplant program in early 2019, received RC as depicted above. The significant difference observed in baseline characteristics between these groups is a bit puzzling. However, we believe that it is partially attributed to selecting less-complicated patients in the initial stages of a novel transplant program. Nevertheless, we believe that this limitation also highlights this study’s strengths and merit. While, for any reason, patients in the DEC groups were older, had higher rates of pretransplant diabetes, as well as diabetes-related microvascular complications, in addition to worse baseline glucose control compared to RC, their glycemic outcomes in this study were, for the most part, non-inferior or even superior compared to those undergoing RC. Furthermore, the significant differences in the patients’ basic characteristics, and particularly in their glycemic control, was mitigated by the mixed model analysis, which reflected changes in glycemic control within each individual patient.

In conclusion, the incorporation of a DEC service among renal recipients is associated with improved glycemic outcomes. Larger, well-powered controlled prospective studies are required to assess the full potential of intensive endocrine care in renal transplant patients as well as other solid-organ recipients.

## Figures and Tables

**Table 1:** Baseline characteristics.

	All (n = 113)	Dedicated Endocrine Care (n = 53)	Routine Care (n = 60)	*p*-Value, CI
Age (mean, SD)	58.2 ± 11.8	61.2 ± 9.7	55.4 ± 12.9	*p* = 0.009, CI (−10.1, −1.5)
Male sex, n (%)	81 (71.7)	42 (79.2)	39 (65)	*p* = 0.09
Pretransplant diabetes, n (%)	57 (50.4)	48 (90.6)	9 (15)	*p* < 0.001
Diabetic nephropathy, n (%)	45 (39.8)	38 (71.7)	7 (11.7)	*p* < 0.001
Diabetic retinopathy, n (%)	21 (18.6)	17 (32.1)	4 (6.7)	*p* < 0.001
Diabetic neuropathy, n (%)	15 (13.3)	13 (24.5)	2 (3.3)	*p* < 0.001
Ischemic heart disease, n (%)	35 (31)	24 (45.3)	11 (18.3)	*p* = 0.002
Peripheral vascular disease, n (%)	6 (5.3)	4 (7.5)	2 (3.3)	0.32
Stroke, n (%)	5 (4.4)	4 (7.5)	1 (1.7)	0.13
Glucose level (mean, SD)	141.4 ± 64.1	168.4 ± 80.6	117.4 ± 29.1	*p* < 0.001 CI (−74.3, −27.6)
HbA1C (mean, SD)	6.1 ± 1.3	6.7 ± 1.3	5.3 ± 1.3	*p* < 0.001 CI (−1.9, −1)

**Table 2:** Glycemic control during post-transplant hospitalization.

	All (n = 113)	Dedicated Endocrine Care (n = 53)	Routine Care (n = 60)	*p*-Value, CI
Below-range BG (<110 mg/dL) (n, %)	442 (17.2)	201 (12.9)	241 (23.6)	*p* < 0.001
Hypoglycemia (<70 mg/dL) n (%)	34 (1.3)	22 (1.4)	12 (1.2)	*p* = 0.6
Time in range ^#^ n (%)	560 (21.7)	324 (20.8)	236 (23.1)	*p* = 0.18
Hyperglycemic insulin response ^##^ n (%)	571 (61.1)	457 (66.4)	114 (46.3)	*p* < 0.001

^#^ Glucose level between 140 and 180 mg/dL, calculated as percentage of measurements. ^##^ Calculated as the fraction of short-acting insulin administered when glucose level ≥ 200 mg/dL.

**Table 3:** Short-term transplant-related complications.

	All (n = 113)	Dedicated Endocrine Care (n = 53)	Routine Care (n = 60)	*p*-Value
Readmission *, n (%)	25 (22.1)	11 (20.8)	14 (23.3)	0.74
Infection *, n (%)	31 (27.4)	14 (26.4)	17 (28.3)	0.82
Rejection, n (%)	16 (14.2)	6 (11.3)	10 (16.7)	0.42
Graft loss, n (%)	7 (6.2)	2 (3.8)	5 (8.3)	0.44

* Within 30 days post-transplantation.

**Table 4:** Daily steroid dosing.

Daily Methylprednisolone Dosage (mg) #	All	Dedicated Endocrine Care Group	Routine Care Group	*p*-Value
Day 1	200 (100–250)	200 (100–200)	200 (106.2–250)	*p* = 0.06
Day 2	100 (100–160)	160 (100–160)	100 (100–160)	*p* = 0.046
Day 3	120 (25–120)	120 (60–120)	25 (25–120)	*p* = 0.009
Day 4	80 (25–80)	80 (25–80)	25 (25–80)	*p* = 0.037
Day 5	25 (25–25)	25 (25–25)	25 (25–25)	*p* = 0.6
Day 6	25 (25–25)	25 (25–25)	25 (25–25)	*p* = 0.69
Day 7	25 (0–25)	25 (0–25)	25 (25–25)	*p* = 0.074
Day 8	0 (0–25)	0 (0–25)	25 (0–25)	*p* = 0.045

# Data are presented in Median (IQR). Prednisone dosage was converted to Methylprednisolone in equivalent dosing.

**Table 5:** Daily glucose coefficient of variation (COV).

	All	Dedicated Endocrine Care Group	Routine Care Group	*p*-Value, CI
Day 1	0.18 ± 0.11	0.18 ± 1	0.19 ± 0.12	*p* = 0.86, CI (−0.04, 0.05)
Day 2	0.2 ± 0.12	0.21 ± 0.11	0.19 ± 0.13	*p* = 0.65, CI (−0.06, 0.04)
Day 3	0.23 ± 0.13	0.25 ± 0.13	0.2 ± 0.14	*p* = 0.11, CI (−0.1, 0.01)
Day 4	0.27 ± 0.15	0.3 ± 0.15	0.24 ± 0.15	*p* = 0.06, CI (−0.12, 0.03)
Day 5	0.27 ± 0.13	0.27 ± 0.14	0.27 ± 0.11	*p* = 0.98, CI (−0.06, 0.06)
Day 6	0.3 ± 0.21	0.32 ± 0.15	0.28 ± 0.28	*p* = 0.44, CI (−0.13, 0.06)
Day 7	0.28 ± 0.14	0.27 ± 0.12	0.3 ± 0.16	*p* = 0.35, CI (−0.04, 0.1)
Day 8	0.26 ± 0.15	0.25 ± 0.13	0.26 ± 0.17	*p* = 0.75, CI (−0.07, 0.1)

## Data Availability

The data presented in this study are available on request from the corresponding author due Chaim Sheba Medical Center privacy regulations. Upon reasonable request and with the approval of the corresponding author, the data can be made available for legitimate research purposes.

## Abbreviations

ADA	American Diabetes Association
BG	Blood glucose
CV	Coefficient of variation
DEC	Dedicated endocrine care
DM	Diabetes mellitus
EPTH	Early post-transplant hyperglycemia
IQRs	Interquartile ranges
PTDM	Post-transplant diabetes mellitus
RC	Routine care
SDs	Standard deviations
TIR	Time in range
